# Stimulation of osteogenic differentiation in human osteoprogenitor cells by pulsed electromagnetic fields: an in vitro study

**DOI:** 10.1186/1471-2474-11-188

**Published:** 2010-08-23

**Authors:** Justus HW Jansen, Olav P van der Jagt, Bas J Punt, Jan AN Verhaar, Johannes PTM van Leeuwen, Harrie Weinans, Holger Jahr

**Affiliations:** 1 Department of Orthopaedics, Erasmus University Medical Center, P.O. Box 2040, 3000 CA Rotterdam, The Netherlands; 2 Department of Internal Medicine, Erasmus University Medical Center, P.O. Box 2040, 3000 CA Rotterdam, The Netherlands; 3 Department of Surgery, Albert Schweitzer Hospital, P.O. Box 444, 3300 AK Dordrecht, The Netherlands

## Abstract

**Background:**

Although pulsed electromagnetic field (PEMF) stimulation may be clinically beneficial during fracture healing and for a wide range of bone disorders, there is still debate on its working mechanism. Mesenchymal stem cells are likely mediators facilitating the observed clinical effects of PEMF. Here, we performed in vitro experiments to investigate the effect of PEMF stimulation on human bone marrow-derived stromal cell (BMSC) metabolism and, specifically, whether PEMF can stimulate their osteogenic differentiation.

**Methods:**

BMSCs derived from four different donors were cultured in osteogenic medium, with the PEMF treated group being continuously exposed to a 15 Hz, 1 Gauss EM field, consisting of 5-millisecond bursts with 5-microsecond pulses. On culture day 1, 5, 9, and 14, cells were collected for biochemical analysis (DNA amount, alkaline phosphatase activity, calcium deposition), expression of various osteoblast-relevant genes and activation of extracellular signal-regulated kinase (ERK) signaling. Differences between treated and control groups were analyzed using the Wilcoxon signed rank test, and considered significant when p < 0.05.

**Results:**

Biochemical analysis revealed significant, differentiation stage-dependent, PEMF-induced differences: PEMF increased mineralization at day 9 and 14, without altering alkaline phosphatase activity. Cell proliferation, as measured by DNA amounts, was not affected by PEMF until day 14. Here, DNA content stagnated in PEMF treated group, resulting in less DNA compared to control.

Quantitative RT-PCR revealed that during early culture, up to day 9, PEMF treatment increased mRNA levels of bone morphogenetic protein 2, transforming growth factor-beta 1, osteoprotegerin, matrix metalloproteinase-1 and -3, osteocalcin, and bone sialoprotein. In contrast, receptor activator of NF-κB ligand expression was primarily stimulated on day 14. ERK1/2 phosphorylation was not affected by PEMF stimulation.

**Conclusions:**

PEMF exposure of differentiating human BMSCs enhanced mineralization and seemed to induce differentiation at the expense of proliferation. The osteogenic stimulus of PEMF was confirmed by the up-regulation of several osteogenic marker genes in the PEMF treated group, which preceded the deposition of mineral itself. These findings indicate that PEMF can directly stimulate osteoprogenitor cells towards osteogenic differentiation. This supports the theory that PEMF treatment may recruit these cells to facilitate an osteogenic response in vivo.

## Background

Pulsed electromagnetic field (PEMF) stimulation may be clinically beneficial in the treatment of fracture healing, especially in non-unions [1-3]. There are indications that PEMF might also be effective in the treatment of osteoporosis [4-6]. While there is a relatively frequent clinical use of electromagnetic stimulation, current evidence from randomized trials is insufficient to conclude a benefit of this treatment modality [7]. Although more knowledge on PEMF-induced effects is becoming available, the underlying cellular mechanisms remain poorly understood.

Likely candidates that might facilitate a stimulatory effect of PEMF in fracture healing are the osteoblasts, or their precursors, the mesenchymal stem cells (MSCs). Aaron et al. suggested that PEMF-enhanced differentiation of mesenchymal stem cells is most likely responsible for the increase in extracellular matrix synthesis and bone maturation [8]. Recent studies indicate that progenitor cells may migrate into bone fracture sites and initiate osteogenic lineage commitment [9]. However, little is known about direct PEMF-induced effects on osteoprogenitor cells as the most likely cell population contributing to the osteogenic response [10-12]. Only recently, Tsai et al. demonstrated a modulating role of PEMF stimulation in MSC osteogenesis [11]. Furthermore, Sun et al. postulated that PEMF exposure could enhance bone marrow mesenchymal stem cells proliferation [12].

To induce a biological response, translation of the electromagnetic signal into a biochemical signal is obligatory. Various, albeit somewhat conflicting, effects of PEMF on transcriptional level, cell proliferation and differentiation have been reported in osteoblasts [13-19]. Multiple studies report positive effects of PEMF on mineralization in osteoblast-like cell cultures [20-22]. This supports findings of in vivo studies which show an increase of mineral apposition rate after PEMF treatment [23,24]. Besides PEMF-induced effects on cellular differentiation, there is increasing evidence suggesting that effects of electromagnetic stimulation are also dependent on cellular maturation stage [8,20,25,26]. Aaron et al. report a temporal stimulation in the mesenchymal stage of endochondral bone development, essential for accelerated bone formation. Additionally, Diniz et al showed that PEMF had a stimulatory effect on the osteoblasts in the early stages of culture, which increased bone tissue-like formation, but decreased bone tissue-like formation in the mineralization stage.

Although many factors are known to be involved in bone growth and repair, the transforming growth factor beta (TGF-β1) family of proteins, including bone morphogenetic proteins (BMPs), are of particular interest due to their well-recognized osteogenic potential [27]. Exogenous BMPs are currently being clinically used to treat non-union fractures [28,29]. PEMF-induced up-regulation of BMP-2 and -4 mRNA has been demonstrated in rat osteoblasts [30]. Additionally, PEMF-enhanced effects of BMP-2 treatment on osteoblastic cell differentiation has been shown in both rat osteoblastic cells and human MSCs [31,32]. Another important bone remodeling system is based on the interaction between osteoblast and osteoclast, regulated by osteoprotegerin (OPG) and receptor activator of NF-κB ligand (RANKL). In particular, the OPG/RANKL ratio is considered to be the dominant regulator, while an increased ratio will result in decreased osteoclastogenesis and thus have an osteoprotective effect [33]. Chang et al. showed PEMF-induced up-regulation of OPG while RANKL mRNA expression was down-regulated, resulting in an increased OPG/RANKL ratio [14].

Matrix metalloproteinases (MMPs) are enzymes responsible for the proteolytic degradation of extracellular matrix components such as collagen that also play a crucial role in bone remodeling. Previous studies have shown that expression of MMPs in osteoblasts can be induced by a variety of extracellular stimuli like e.g. mechanical loading [34-36]. However, the effect of PEMF on MMP expression in MSCs has not yet been studied.

While the majority of reported PEMF-induced effects were studied in osteoblasts, findings in literature indicate that MSCs could be a more relevant cell source in vivo. We hypothesize that PEMF directly stimulates osteoprogenitors towards osteogenic differentiation. The current study investigates the effects of PEMF on human bone marrow-derived stromal cells (BMSCs) during subsequent stages of osteogenic differentiation. DNA amounts, differentiation, and mineralization were monitored to assess maturation stage. To address which mediators play a role in PEMF exerted effects in BMSCs, mRNA expression levels of osteoblast-relevant genes were tested during the differentiation process. As PEMF induced effects on proliferation may be cell type dependent, DNA amounts were monitored in both BMSCs and already committed human fetal pre-osteoblasts (SV-HFOs). The extracellular signal-regulated kinase-1/2 (ERK) signaling pathway is crucial for osteoblast function and differentiation [37-39]. As ERK signaling is known to play an important role in mechanotransduction [36,40,41], its possible involvement in PEMF-mediated effects was assessed.

## Methods

### Cell culture

Human bone marrow stromal cells (BMSCs) were isolated from bone marrow aspirates obtained during total hip revision surgery after approval by the local ethical committee (MEC2004-322). Bone marrow aspirates were taken from the greater trochanter. Heparinized aspirates were seeded at a density of 30 to 90 × 10^6 ^nucleated cells per T175 flask. After 24 h, non-adherent cells and cell debris were washed out. hBMSCs were further expanded in low-glucose Dulbecco's modified Eagle medium (DMEM) (Gibco, Paisly, UK) with 10% fetal calf serum (FCS) from a pre-selected batch to maintain the multipotential capacities of the cells, 50 μg/mL of gentamicin, and 1.5 μg/mL of Fungizone (all Invitrogen, Carlsbad, CA) and 0.1 mM of L-ascorbic acid 2-phosphate and 1 ng/mL of fibroblast growth factor (Instruchemie B.V., Delfzijl, the Netherlands). Cells were cultured at 37°C under humidified conditions and 5% carbon dioxide (CO_2_). Medium was changed twice a week. When cultures neared confluence, they were trypsinized using 0.05% trypsin and replated at a density of 2000 cells/cm^2^. Cells from the second to the fourth passage were used for experimental purpose [42]. After expansion, cells were seeded onto six-well plates at an initial density of approximately 1 × 10^5 ^cells per well, and cultured up to 14 days in osteogenic medium: DMEM containing 10% FCS, 0.1 mM ascorbic acid, 1 μM dexamethasone (Sigma, St. Louis, MO), and 10 mM β-glycerophosphate (Sigma). The BMSCs from four different donors were analyzed after 1, 5, 9, or 14 days of culture in osteogenic media. All cultures were fully mineralized after 14 days.

SV40-immortalized human fetal pre-osteoblasts (SV-HFO) were seeded similarly to the BMSCs and cultured in αMEM medium without phenol red (Gibco) and 2% FCS [43,44]. To induce osteoblastic differentiation, 1 μM dexamethasone and 10 mM β-glycerophosphate was added to the medium. Cells were induced towards osteoblastic differentiation as described earlier and harvested after 7, 14, or 21 days after which they were fully mineralized. To exclude a possible influence of serum on PEMF-induced effects, additional experiments with varying percentage FCS (2 or 10%) were conducted with emphasis on DNA amount.

### Pulsed electromagnetic field

Pulsed electromagnetic fields (PEMF) were generated by a commercially available bone-healing device (Orthopulse^® ^II, IMD, currently distributed by OSSATEC^®, ^Uden, The Netherlands). The coils of the device were supported by an acrylic frame in which six-well plates were placed. Treated cell cultures were continuously exposed to a 15 Hz, 1 Gauss EM field, consisting of 5-millisecond bursts with 5-microsecond pulses.

### Analysis

*DNA, alkaline phosphatase activity and mineralization - *For characterization, DNA amount, alkaline phophatase (ALP) activity and calcium deposition as a measure for mineralization were determined. Cell lysates in 0.1% PBS-Triton X-100 were treated with heparin and RNase A (50 mg/ml in PBS) for 30 minutes at 37°C. DNA content was measured according to the ethidium bromide method by Karsten and Wollenberger [45]. Calcium deposition into the extracellular matrix was determined after overnight extraction with HCl, using the Sigma calcium assay according to manufacturer's description. Alkaline phosphatase activity was determined by the colorimetric method of Lowry et al.[46]. Results were adjusted for DNA content of the corresponding cell lysates.

#### Western blotting

On day 14, SV-HFOs or BMSCs were lysed in buffer (200 μl/well) containing 25 mM HEPES, 1% Triton X-100, 1% deoxycholate, 0.1% SDS, 0.5 M NaCl, 5 mM EDTA, 50 mM NaF, 1 mM PMSF, 1 mM Na_3_VO_4_, 10 μg/ml leupeptin, and 2 mM β-glycerophosphate, and harvested with a cell scraper. After centrifugation at 11000× *g *for 15 minutes, supernatant was collected and stored at -80°C until further use. Protein levels were determined using BCA protein assay kit (Pierce, Rockford, IL). Cell lysate (10 μg protein/lane) was separated by SDS-PAGE and transferred to Hybond+ nitrocellulose membranes (Amersham Pharmacia Biotech, Piscataway, NJ). After overnight blocking in TBS + 0.1% Tween 20 containing 5% (w/v) bovine serum albumine, membranes were incubated with monoclonal anti-ERK1/2-P antibodies (1:2000, Cell Signaling, Beverly, MA) for 3 h at room temperature and detected using horseradish peroxidase coupled secondary antibodies and the ECL detection system (Amersham Pharmacia Biotech). After exposure to Kodak HR film, immunoreactive bands at 42 kD and 44 kD were quantified using Quantity One (Bio-Rad) software. Finally, all blots were stripped and blocked again for incubation with monoclonal antibodies against ERK1/2 (1:2000, Cell Signaling, Beverly, MA). Total ERK1/2 levels were used as loading controls.

#### Quantification of gene expression

BMSCs were collected in 500 μl RNA-Bee™(TEL-TEST, Friendswood, TX, USA) per well. RNA was purified, quantified and reverse transcribed as described earlier [36]. Real-time PCR conditions and normalization to GAPDH, which was stably expressed throughout the experiments, were adopted from Das et al. [47,48]. TaqMan^® ^hydrolysis probe assays are reported by Mandl et al. [49], while remaining primer and probe nucleotide sequences (Table 1) were designed using PrimerExpress2.0. All Taqman assays were performed in triplicates in 96-well optical plates using qPCR™ Core Kit (Eurogentec, Maastricht, The Netherlands) and similar amplification efficiencies between assays were verified by cDNA serial dilutions (data not shown). Specificity of listed oligonucleotides was checked by BLASTN^® ^(Basic Local Alignment Search Tool) against the human RefSeq RNA database at NCBI and verified by standard agarose gel electrophoresis.

**Table 1:** List of genes and primer and probe nucleotide sequences.

Gene	Primer^#^	Reference	Primer (5'-3')
OC	F	NM_199173.3	GAAGCCCAGCGGTGC
		
	R		CACTACCTCGCTGCCCTCC
		
	FAM		TGGACACAAAGGCTGCACCTTTGCT

OPG	F	NM_002546.3	GCAGCGGCACATTGGAC
		
	R		CCCGGTAAGCTTTCCATCAA
		
	FAM		TGCTAACCTCACCTTCGAGCAGCTTCGTA

RANKL	F	NM_003701.2	CGTTGGATCACAGCACATCAG
		
	R	NM_033012.2	GCTCCTCTTGGCCAGATCTAAC
		
	FAM		CAGAGAAAGCGATGGTGGATGGCTCAT

BMP2	F	NM_001200.2	AACACTGTGCGCAGCTTCC
		
	R		CTCCGGGTTGTTTTCCCAC
		
	FAM		CCATGAAGAATCTTTGGAAGAACTACCAGAAACTG

TGF-β1	F	NM_000660.3	CGAGCCTGAGGCCGACTAC
		
	R		AGATTTCGTTGTGGGTTTCCA
		
	FAM		-

SPP1	F	NM_001040060.1	CTCAGGCCAGTTGCAGCC
		
	R	NM_001040058.1	CAAAAGCAAATCACTGCAATTCTC
		
	FAM	NM_000582.2	AAACGCCGACCAAGGAAAACTCACTACC

SPARC	F	NM_003118.2	ATCTTCCCTGTACACTGGCAGTTC
		
	R		CTCGGTGTGGGAGAGGTACC
		
	FAM		CAGCTGGACCAGCACCCCATTGAC

IBSP	F	NM_004967.2	TGCCTTGAGCCTGCTTCC
		
	R		GCAAAATTAAAGCAGTCTTCATTTTG
		
	FAM		CTCCAGGACTGCCAGAGGAAGCAATCA

MMP-1	F	NM_002421	CTCAATTTCACTTCTGTTTTCTG
		
	R		CATCTCTGTCGGCAAATTCGT
		
	FAM		CACAACTGCCAAATGGGCTTGAAGC

MMP-3	F	NM_002422	TTTTGGCCATCTCTTCCTTCA
		
	R		TGTGGATGCCTCTTGGGTATC
		
	FAM		AACTTCATATGCGGCATCCACGCC

^# ^Primer = Forward (F), Reverse (R), FAM-labeled TaqMan^® ^probe (FAM). All assays are TaqMan^® ^assays except for TGF- β1 (SYBRGreen assay).

#### Statistics

Experiments were performed using BMSCs from four different donors. Within each donor, conditions were tested in triplicate (n = 3). Data are presented as means from four donors for each condition ± standard deviation. Statistical analysis was performed on all triplicates from the four donors. To take donor variability into consideration, experimental groups were analyzed using the Wilcoxon signed rank test, and considered significant when p < 0.05. For SV-HFOs, experiments were performed in triplicate (n = 3). Differences were analyzed using the Mann-Whitney U test and considered significant when P < 0.05.

## Results

**Figure 1:**
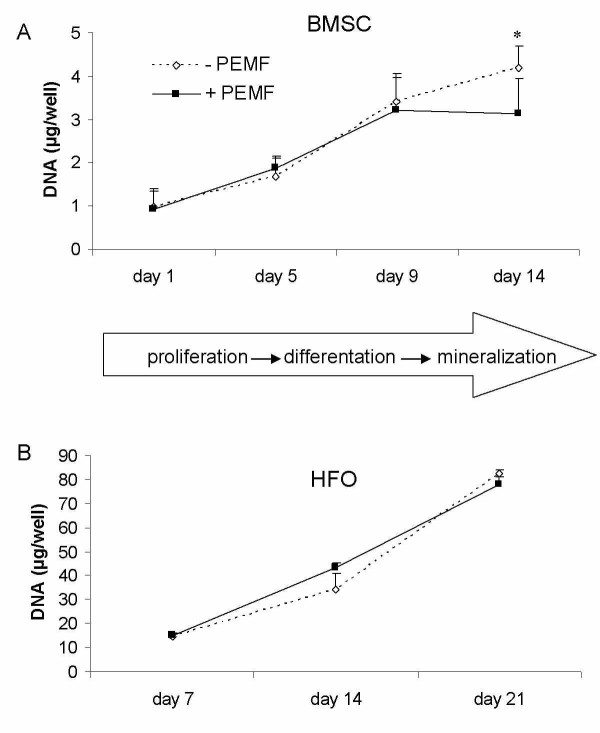
**Effect of PEMF stimulation on DNA amounts in two different cell types**. A) human bone marrow derived stromal cells, obtained from 4 different donors (n = 3 per donor), B) human fetal pre-osteoblasts (n = 3). Cells were cultured in osteogenic medium to full mineralization. Significant differences due to PEMF are marked * (p < 0.05, Wilcoxon signed rank test).

**Figure 2:**
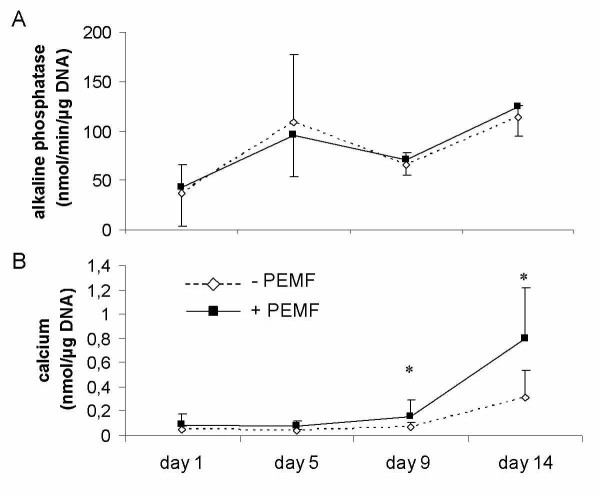
**Effect of PEMF stimulation on osteogenic differentiation of human BMSCs**. Figure shows alkaline phosphatase activity (A) and calcium deposition in the extracellular matrix (B) of these cells. Cells were obtained from 4 differenent donors (n = 3 per donor). Significant differences due to PEMF are marked * (p < 0.05, Wilcoxon signed rank test).

Cultured in osteogenic medium, human bone marrow stromal stem cells (BMSCs) and fetal pre-osteoblasts (SV-HFOs) showed a gradual increase in DNA content over time (figure 1). Both cell types were cultured up to full mineralization. In BMSCs, PEMF treatment did not significantly alter DNA amount until day 14, when DNA contents per well further increased in the control group but not in the PEMF-treated group (figure 1A). In SV-HFOs, no effect of PEMF on DNA content was observed at any differentiation stage (figure 1B), regardless of the percentage of serum used (data not shown). In general, alkaline phosphatase (ALP) activity increased around day 5, both in the PEMF treated cells and controls. However, the time point at which peak activity was reached, varied between BMSCs from different donors causing high standard deviation at day 5. At day 14, a further increase in ALP per DNA was observed. In general, ALP activity was not significantly affected by PEMF treatment (figure 2A). PEMF treatment did not affect the timing of matrix mineralization, starting around day 9 in both the control and PEMF treated groups. At day 9 and 14, mineralization (measured as amount of calcium normalized to DNA) was stronger induced in the PEMF treated group (figure 2B).

**Figure 3:**
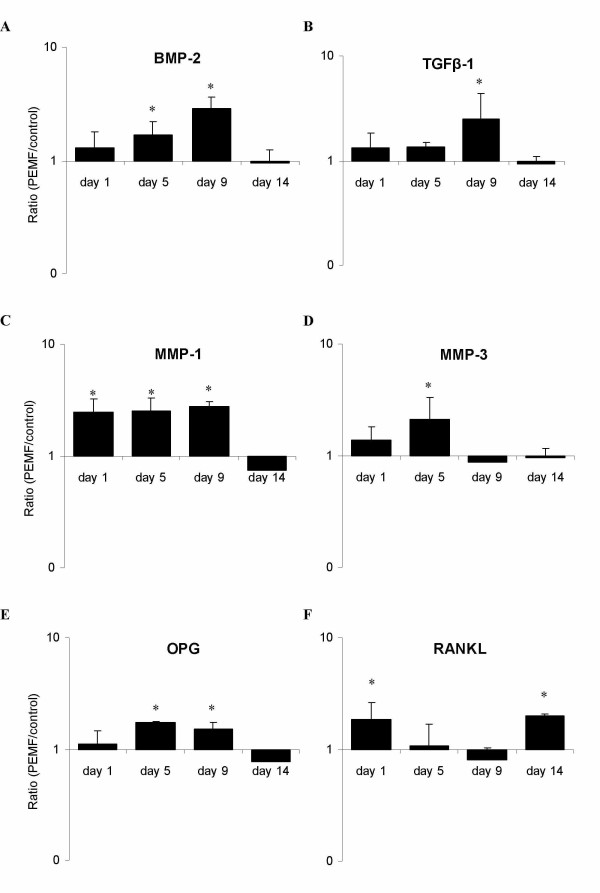
**Effect of PEMF on osteoblast-relavant genes in human BMSCs**. Gene expression ratios of selected genes plotted as expression in PEMF-treated condition relative to non-treated control. Targeted genes are A) bone morphogenetic protein 2 (BMP-2); B) transforming growth factor beta 1 (TGF-β1); C) osteoprotegrin (OPG); D) receptor activator of NF-kappa-B ligand (RANKL); E) matrix metalloproteinase 1 (MMP-1); F) matrix metalloproteinase 3 (MMP-3). Significant differences due to PEMF are marked * (p < 0.05), n = 4.

**Figure 4:**
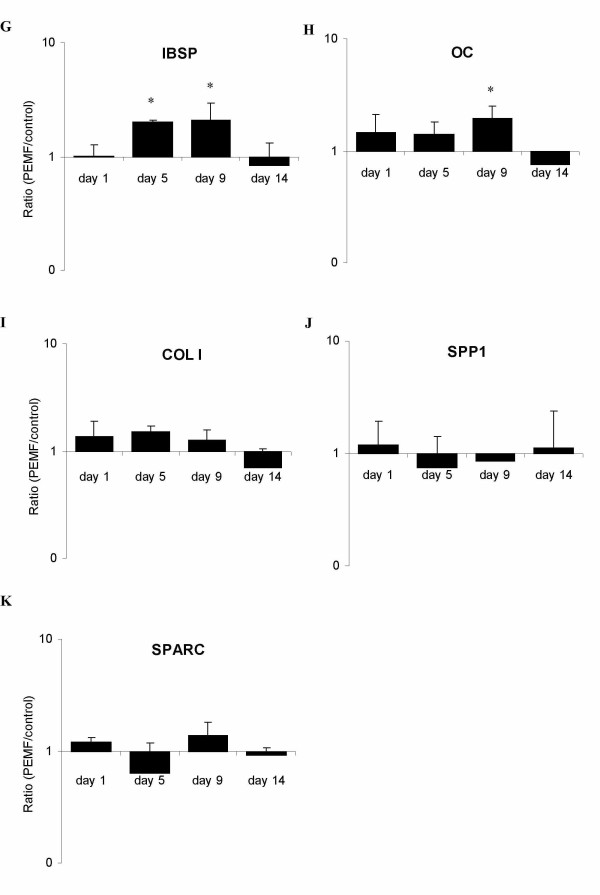
**Effect of PEMF on osteoblast-relavant genes in human BMSCs**. Gene expression ratios of selected genes plotted as expression in PEMF-treated condition relative to non-treated control. Targeted genes are G) collagen 1 (COL1); H) osteocalcin (OC); I) bone sialoprotein 1 (IBSP); J) osteonectin (SPARC); K) osteopontin (SPP1). Significant differences due to PEMF are marked * (p < 0.05), n = 4.

**Figure 5:**
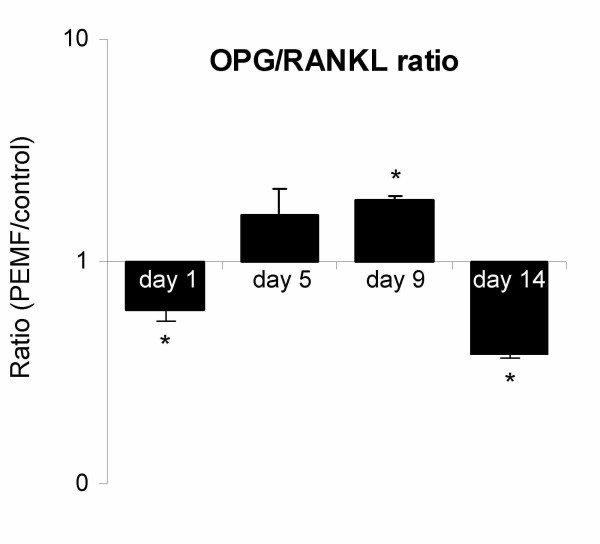
**Effect of PEMF on *OPG/RANKL *expression ratio in human BMSCs**. Gene expression ratios of OPG/RANKL plotted as expression in PEMF-treated condition relative to non-treated control. Significant differences due to PEMF are marked * (p < 0.05), n = 4.

Expression levels of osteoblast-relevant genes in human BMSCs ranged from highly abundant collagen I (*COL I*, C_T_≈ 17) to weakly expressed receptor activator of NF-κB ligand (*RANKL*, C_T_≈ 33). In general, highest expression levels were observed just before and around the onset of mineralization (day 5 and 9). The reference gene GAPDH remained stably expressed at all tested days throughout the experiment. Bone morphogenetic protein 2 (*BMP-2*) was most responsive to PEMF, with a maximum increase in expression of 3.5-fold over control on day 9 of culture (figure 3A). Expression of transforming growth factor-beta (*TGF-β1*) was 2.5-fold up-regulated upon PEMF treatment at the same day (figure 3B). Interestingly, neither gene was affected by PEMF at the later stages of mineralization (day 14). Matrix metalloproteinase (*MMP*) -1 and -3 expression levels were both up-regulated by PEMF (figure 3C + D): *MMP-1 *was found to be constantly up-regulated until day 9, reaching a 2.8-fold expression, while *MMP-3 *was up-regulated until day 5 (2.1-fold). Again, no significant effect of PEMF was observed on day 14 for both MMPs. Other tested genes that were up-regulated by PEMF were osteoprotegerin (*OPG*: 1.7-fold; figure 3E), bone sialoprotein (*IBSP*: 2-fold; figure 4G), and osteocalcin (*OC*: 2-fold; figure 4H). However, on day 14 the stimulatory effect of PEMF was no longer apparent for the above-mentioned genes. In contrast, *RANKL *expression, which was insensitive to PEMF treatment earlier in BMSC culture (day 5 and 9), was stimulated on day 14 (figure 3D). The OPG/RANKL ratio was calculated to indicate possible effects on osteoclastogenesis. Until day 9, the ratio increased due to PEMF. At day 14, however, the ratio was reversed again (figure 5). Expression of collagen type I (*COL1*; figure 4I), osteopontin (*SPP1*; figure 4J) and osteonectin (*SPARC*; figure 4K) was not significantly regulated by PEMF stimulation, although a stimulatory trend was observed for collagen I from day 1 to day 9 (p-values of 0.086 on day 1 and 0.074 on day 5).

**Figure 6:**
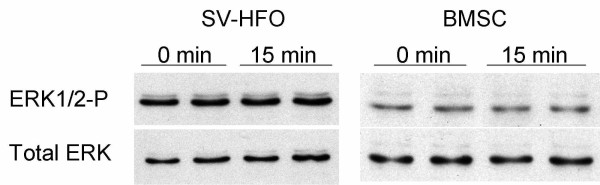
**Western blot showing activated, phosphorylated ERK (ERK1/2-P) next to total ERK (ERK1/2) specific signals as loading control**. Human fetal pre-osteoblasts (SV-HFO) and bone marrow stromal cells (BMSC) were cultured up to day 14 prior to PEMF treatment. PEMF exposure of 15 minutes was compared with control (0 minutes of PEMF).

To address a potential ERK activation upon PEMF treatment, SV-HFOs and BMSCs were exposed to 15 minutes of PEMF on day 14. During this stage of mineralization, PEMF-induced signaling seems to be ERK-independent as no significant differences in phosphorylation could be detected. Total ERK levels were similar between the PEMF treated and control groups, indicating equal loading (figure 6).

## Discussion

We found that PEMF stimulation of osteogenic differentiating BMSCs enhanced mineralization after 14 days of PEMF exposure, compared to the control. The ability of PEMF to promote mineralization has been described in osteoblasts [31], but not yet been demonstrated in BMSCs. Tsai et al. recently showed a modulating role of PEMF in similar cells [11]. In contrast to our findings, no effect of PEMF on mineralization was observed. However, by only using a histochemical staining on day 14 and 28, subtle changes in mineralization may have been missed in their setup.

Interestingly, DNA amounts did not further increase in the PEMF treated BMSC culture after day 9, while they did in the control group. PEMF seemed to induce differentiation at the expense of proliferation - a generally accepted principle that has been demonstrated using osteoblasts, as well as human mesenchymal stem cells [11,14,20]. The osteogenic stimulus of PEMF was further confirmed by the up-regulation of several osteogenic marker genes (e.g. *BMP-2, OC, IBSP*) in the PEMF treated group, which preceded the deposition of mineral (Ca^2+^) itself. These findings strongly suggest that PEMF stimulation enhanced osteogenic differentiation in BMSCs.

PEMF exposure did not affect DNA content in BMSCs until day 14. Here, the increase in DNA content halted in the PEMF treated BMSC group, parallel with the onset of mineralization. This could well be the result of inhibition of proliferation, a process required to proceed through the maturation process [50]. Another explanation for this finding is PEMF-induced apoptosis, although a previous study by Nikolova et al. points out that, while PEMF exposure transiently may affect the transcript level of genes related to apoptosis and cell cycle control [51], no detectable changes of cell physiology were found.

Data on proliferative effects of PEMF described in literature in osteoblasts vary considerably: the maximum effect of PEMF exposure on cell proliferation appears to depend on the percentage of FCS in the medium [52], cell density [53], cell type [54], differentiation stage and characteristics of PEMF [55]. In order to verify our findings of PEMF on cell number in BMSCs, we examined PEMF effects on DNA amount in various human cell types, including SV-HFOs (Figure 1B) and human umbilical vein endothelial cells (not shown). No significant effect on DNA content was found, despite low initial cell density or the presence of 10% serum, as described by others [13,15,52,54]. Additionally, the variety in characteristics of PEMF devices used in separate studies may contribute to different findings between research groups. Matsunaga et al. have shown PEMF-induced osteogenesis to be dependent on the intensity and pulse duration of the stimulation [55].

In our culture model, the onset of mineralization on day 9 seemed to be an important hallmark with respect to regulation of gene expression by PEMF. Most genes were up-regulated in the period preceding and during the onset of mineralization. *TGF-β1 *expression, which was induced by PEMF up to day 9, is also elevated in osteoblasts subjected to mechanical strain [56], and an important role of this cytokine in fracture repair has been suggested [57]. Expression of *BMP-2 *was also gradually stimulated upon PEMF exposure up to 3.5 fold until the onset of mineralization. BMPs have been used successfully in the clinical setting to help treat non-union fractures, often in combination with bone grafts [58], and one might that PEMF's reported positive effects may be secondary to an endogenous stimulation of osteoinductive cytokines of the TGF-beta superfamily (e.g., BMP-2, -4, -7) *in vivo*. *In vitro *studies have demonstrated osteogenic differentiation of rat primary osteoblastic cells and human MSCs after treatment with BMP-2, the latter being further enhanced upon PEMF stimulation [10,31]. Several BMPs have been shown to dominantly induce osteogenic differentiation of MSCs independent of other known stimuli [59]. Moreover, BMP-2 strongly induces osteogenic transdifferentiation of other progenitors, like myogenic cells [60]. Increased *BMP-2 *mRNA levels after PEMF treatment have been shown in chick embryonic calvaria and rat osteoblasts, indicating that PEMF indeed mediates endogenous stimulation of BMPs [30,61,62]. Additionally, Wang et al. found increased BMP-2 protein production after stimulation with electric fields [62].

We demonstrated PEMF-induced up-regulation of MMPs in human BMSCs during osteoblastic differentiation, which has not earlier been evaluated in relation to PEMF treatment. Again, these effects were observed until day 9 of culture. The up-regulation of MMPs may result in faster remodeling of collagenous matrix, and is for example observed during increased bone turnover. Typically, both *MMP-1 *and -*3 *and mineralization were induced by mechanical loading in vitro as well [36]. PEMF-induced up-regulation of MMP expression might be indicative of increased matrix remodeling and play a crucial role in the beneficial clinical results of PEMF in treatment of non-unions. Not surprisingly, expression of extracellular matrix marker proteins, like osteocalcin and bone sialoprotein, was found to be up-regulated after PEMF exposure until day 9, too. Although not significantly, collagen type I expression was steadily induced in the early stages of differentiation (p-values of 0.086 on day 1 and 0.074 on day 5), potentially facilitating extracellular matrix synthesis. Heermeier et al. also found enhancement of collagen type I mRNA expression after stimulation with electromagnetic field in human osteoblastic cells, which was induced by TGF-β treatment as well [63].

Our data suggest that the strongest effect of PEMF on BMSCs and stimulation of mineralization is exerted in the period prior to mineralization. This notion is supported by data from Aaron and Ciombor, who investigated the effects of PEMF in a model of endochondral bone formation, and reported that PEMF stimulation in the early stages of mesenchymal maturation was more effective in increasing ossicles' development and mineralization than stimulation in the later stages [8]. The importance of the period prior to mineralization for the eventual extent of mineralization is supported by recent data by Eijken et al., demonstrating control of mineralization by early osteoblastic effects [64]. Interestingly, Tsai et al also observed down-regulation of osteogenic marker genes in PEMF exposed MSCs, after initial up-regulation [11]. Although the underlying mechanism is not clear, this implicates that the effect of PEMF on gene expression is importantly dependent on differentiation stage of the cells. Additionally, we found an increase in *OPG *mRNA expression after PEMF on day 9, while that of *RANKL *was not significantly altered. This resulted in a potentially osteoprotective increase in the OPG/RANKL ratio, which is in agreement with data from murine osteoblasts [14]. However, the expression pattern reversed on day 14.

Alkaline phosphatase increased around day 5. While ALP is an important marker for osteoblast differentiation, we did not observe a significant PEMF induced increase. Tsai et al. demonstrated a PEMF induced increase in ALP activity only on day 7, but not on day 3 and 10 [11]. As we experienced variations of the time point at which peak activity was reached between BMSCs, it is possible that we missed the PEMF induced stimulation of ALP activity.

Nie et al. reported a modulation of ERK activation by PEMF in fibroblasts [18]. ERK signaling is crucial for osteoblast function and differentiation, and has been shown to be activated upon mechanical stimuli in osteoblasts and MSCs. Interestingly, PEMF did not increase ERK phosphorylation in our experimental setup, although culture conditions were identical to those used previously to demonstrate activation of ERK after stretch [65]. This indicates that PEMF and mechanical stimuli may act via different ways of mechanotransduction in differentiating osteoblasts as compared to fibroblasts.

## Conclusions

PEMF exposure of differentiating human BMSCs resulted in early up-regulation of several osteoblast-related genes and enhanced mineralization. These findings indicate that PEMF can directly stimulate mesenchymal stem cells and promote osteogenesis. Differentiation stage, and in particular the onset of mineralization, appeared to be an important hallmark with respect to gene regulation by PEMF. Stimulatory effects were predominantly observed in the pre-mineralization period. These findings support the theory that in vivo PEMF treatment may recruit human bone marrow stromal cells to the osteogenic lineage and use these cells as likely cell pool to facilitate an osteogenic response.

## Competing interests

The authors declare that they have no competing interests.

## Authors' contributions

JHJ conceived of the study, designed the study, carried out the experiments, performed statistical analysis and drafted the manuscript. OPJ participated in the design of the study and carried out the experiments. BJP conceived of the study and helped to draft the manuscript. JAV participated in its design and coordination, and helped to draft the manuscript. JPL participated in the design and coordination, and helped to draft the manuscript. HW conceived of the study, participated in its design and coordination, and helped to draft the manuscript. HJ participated in the study design, developed primer-probe sets, participated in coordination and helped to draft the manuscript.

All authors read and approved the final manuscript.

## Pre-publication history

The pre-publication history for this paper can be accessed here:

http://www.biomedcentral.com/1471-2474/11/188/prepub
